# Exploration of ethnomedicinal plants and their practices in human and livestock healthcare in Haripur District, Khyber Pakhtunkhwa, Pakistan

**DOI:** 10.1186/s13002-021-00480-x

**Published:** 2021-09-08

**Authors:** Zeeshan Siddique, Nasir Shad, Ghulam Mujtaba Shah, Abid Naeem, Liu Yali, Muhammad Hasnain, Arshad Mahmood, Muhammad Sajid, Muhammad Idrees, Ilyas Khan

**Affiliations:** 1 Department of Biosciences, University of Wah, Wah Cantt, Pakistan; 2 Key Laboratory of Silviculture, Collaborative Innovation Center of Jiangxi Typical Trees Cultivation and Utilization, College of Forestry, Jiangxi Agricultural University, Nanchang, 330045 People’s Republic of China; 3 Department of Botany, Hazara University, Mansehra, 21300 Khyber Pakhtunkhwa Pakistan; 4 Key Laboratory of Modern Preparation of Traditional Chinese Medicine, Ministry of Education, State Key Lab of Innovation Drug and Efficient Energy-Saving Pharmaceutical Equipment, Jiangxi University of Traditional Chinese Medicine, Nanchang, 330004 Jiangxi People’s Republic of China; 5 Jiangxi University of Traditional Chinese Medicine, 818 Meiling Road, Nanchang, 330006 People’s Republic of China; 6 Science and Technology College, Jiangxi University of Traditional Chinese Medicine, 818 Meiling Road, Nanchang, 330006 People’s Republic of China; 7 State Environmental Protection Key Laboratory of Wetland Ecology and Vegetation Restoration, School of Environment, Northeast Normal University, Changchun, 130117 People’s Republic of China; 8 Soil Science and Plant nutrient Unit, Brunei Agricultural Research Center, Kilanas, BG 1121 Brunei Darussalam; 9 Department of Agriculture, Hazara University, Mansehra, 21300 Khyber Pakhtunkhwa Pakistan; 10 Department of Mathematics, College of Science Al-Zulfi, Majmaah University, Al-Majmaah, 11952 Saudi Arabia

**Keywords:** Ethnobotany, Ethnoveterinary, Human healthcare, Medicinal plants, Livestock, Traditional knowledge, Haripur District, Pakistan

## Abstract

**Background:**

The utilization of plants and plant resources for various ethnobotanical purposes is a common practice in local towns and villages of developing countries, especially in regard to human and veterinary healthcare. For this reason, it is important to unveil and document ethnomedicinal plants and their traditional/folk usage for human and livestock healthcare from unexplored areas. Here we advance our findings on ethnomedicinal plants from Haripur District, Pakistan, not only for conservation purposes, but also for further pharmacological screenings and applied research.

**Methodology:**

Information of ethnomedicinal plants was obtained using a carefully planned questionnaire and interviews from 80 local people and traditional healers (Hakims) in Haripur District, Pakistan, from 2015 to 2017. Informed consent was obtained from each participant before conducting the interview process. Quantitative ethnobotanical indices, such as relative frequency of citation (RFC), use value (UV) and Jaccard index (JI), were calculated for each recorded species. Correlation analysis between the RFC and UV was tested by Pearson’s correlation, SPSS (ver. 16).

**Results:**

A total of 80 plant species (33 herbs, 24 trees, 21 shrubs and 2 climbers) belonging to 50 families were being used in the study area to treat livestock and human diseases. Lamiaceae was the most dominant family with 7 species (8.7%), followed by Fabaceae with 6 species (7.5%), and Moraceae with 5 species (6.2%). Local people used different methods of preparation for different plant parts; among them, decoction/tea (22 species) was the popular method, followed by powder/grained (20 species) and paste/poultice (14 species). It was observed that most of the species (~ 12 to 16 species) were utilized to treat human and livestock digestive system-related problems, respectively. The Jaccard index found that plant usage in two studies (District Abbottabad and Sulaiman Range) was more comparable. Local people mainly relied on folk medicines due to their rich accessibility, low cost and higher efficacy against diseases. Unfortunately, this important traditional knowledge is vanishing fast, and many medicinal plants are under severe threat. The most threats associated to species observed in the study area include Dehri, Garmthun, Baghpur, Najafpur and Pharala.

**Conclusion:**

The study has indicated that local people have higher confidence in the usage of ethnomedicinal plants and are still using them for the treatment of various ailments. Comparative analysis with other studies may strongly reflected the novel use of these plants, which may be due to the deep-rooted and unique socio-cultural setup of the study area. However, awareness campaigns, conservation efforts and pharmacological and applied research are required for further exploration and may be a step in the right direction to unveil prospective pharmaceuticals.

**Supplementary Information:**

The online version contains supplementary material available at 10.1186/s13002-021-00480-x.

## Introduction

Humans have a long history of utilizing plants to fulfill various daily requirements. Plants are used as medicines, food, fodder for livestock and materials to construct houses [1]. The application of medicinal plants and herbs for therapeutic purposes is a global practice, and almost every country has benefitted from their useful therapeutic and medicinal elements [2]. Herbal medicines play a distinctive role from the primitive period until today in healthcare systems. The first ethnomedicinal plant in sub-continent history was recorded in Rigveda during 4500–1600 BC and Ayurveda 2500–600 BC [3]. The concepts of ethnobotanical medicines are thought to have originated from Greece and adopted by Arabs, thereafter learned and spread by Indians and Europeans [4–6]. Medicinal plants are an important part of the conventional healthcare system, as various allopathic drugs are extracted or derived from medicinal plants [7, 8]. The utilization of alternative medicine may increase due to its low costs, higher efficacy and increased faith in herbal remedies. Although allopathic medicines can treat several diseases, they are often more expensive and may have adverse effects, which forces common people to take advantage of herbal medicines, which may have fewer side effects [9]. Scientific investigations on medicinal plants have been underway in various countries due to their vast therapeutic potential and are also used as an alternative therapy in various healthcare systems [10].

Traditional veterinary medicine was first practiced around 1800 B.C. during the age of King Hammurabi of Babylon, who formulated laws and introduced a veterinary fee structure for treating animals [11]. Ethnoveterinary medicine (EVM) is the major source for the treatment of diseases in livestock throughout the world, even today. Humans have used herbal remedies to treat different diseases in domesticated animals since the advent of civilization. It is estimated that medicinal plants, for several centuries, have been widely used as a primary source of prevention and control of livestock diseases [12, 13]. Many studies have been carried out on treating specific ailments in livestock with herbal medicines and their derivatives [14]. Traditional EVM provides affordable therapy and easy accessibility in comparison to western medicines [15].

Pakistan is an agricultural country, and about 80% of its population depends on farming and livestock. Pakistan is the world's fifth-largest milk-producing country because of its high reliance on farming and livestock [16]. About 84% of Pakistan’s population depended on traditional medicine in the early 1950s, and a rapid decrease was recorded in recent years from traditional knowledge, now limited only to remote areas of Pakistan [17, 18]. Resource-poor farmers of Pakistan substantially depend upon traditional medicine because of their minimal access to modern-day healthcare systems and lack of well-developed basic healthcare units in their areas [3]. While much work has been done worldwide on documenting ethnoveterinary practices, in Pakistan, very little attention has been given to documentation of plants used as EVM, and there is an immense need to document this knowledge [19].

While literature has revealed that many ethnobotanical researchers have visited most parts of Pakistan in recent years, but no/less areas has been thoroughly explored regarding the EVM [20]. A similar trend is evident in human medicinal plant inventories, where many researchers and ethnobotanists have visited most parts of Pakistan and contributed to the records [20–26]. Still, much information and traditional knowledge remain to be recorded. The main aim of this study was (1) to document the traditional knowledge of ethnomedicinal plants from Haripur District, Khyber Pakhtunkhwa (Pakistan), an unexplored area which lacks such documentation, (2) to report the traditional folk knowledge, ethnomedicinal plant utilization along with recipes, mode of preparation, parts used, used form in veterinary and human healthcare by local and ethnic communities, (3) to identify potential conservation threats, (4) to compile the data of traditional knowledge of ethnomedicinal plants by using quantitative ethnomedicinal indices like UV, RFC and JI in order to evaluate the most frequently used species and access their matching with other studies published from Pakistan in traditional ethnomedicinal plant utilization. It is hypothesized that studies conducted in surrounding areas may more similar to present study which can be evaluated by JI value; and (5) to provide further research baseline to pharmacologists, phytochemists and conservationists for further research studies.

## Materials and methods

This study was authorized by the Department of Bioscience and Office of Research, Innovation and Commercialization University of Wah (ORIC-UW), Wah Cantt, Pakistan. Informed consent was obtained from each informant before conducting the semi-structured interview process.

The research study was completed in four phases as follows, (1) description of the study area, (2) ethnomedicinal field survey (primary data), (3) plant’s identification and statistical analysis (secondary data) and (4) data compilation/documentation.

### Study area

**Fig. 1:**
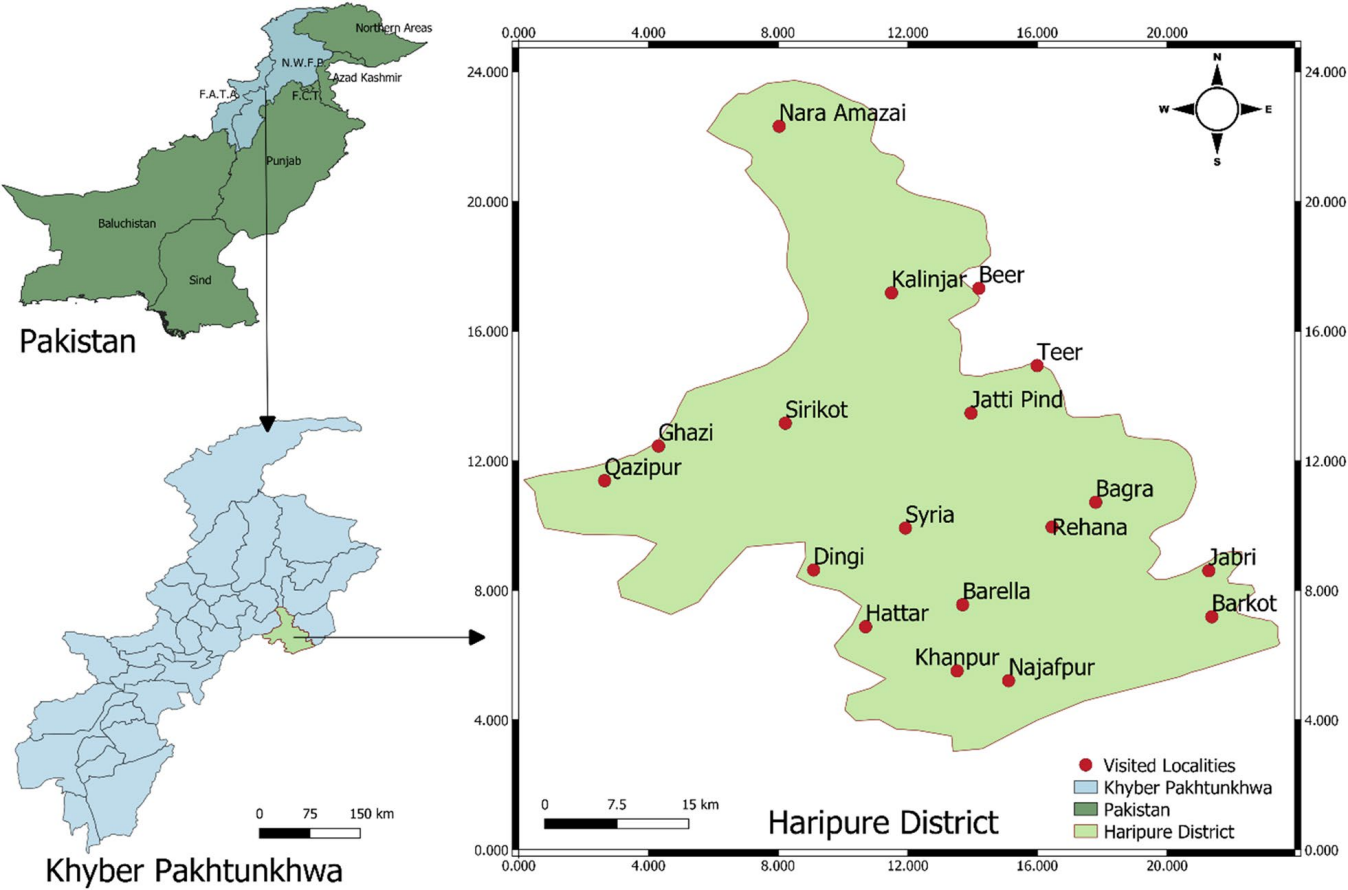
Map of the study area showing sampling site of Haripur District, Pakistan

Haripur District is under the Khyber Pakhtunkhwa province of Pakistan, situated between 33° 44ʹ–34° 22ʹ N latitude and 72°–35ʹ to 73°–15ʹ E longitude, at approximately 610 m above the sea level (Fig. 1). The district's total area is 1725 km^2^, divided into sub-districts (Haripur, Khanpur and Ghazi) and subdivided into 44 Union Councils. Haripur District has distinct geographical significance as its boundaries touch Districts Abbottabad, Mansehra, Attock, Torghar, Swabi, Buner, Rawalpindi (Punjab province) and the capital of Pakistan (Islamabad) [27]. According to the National Institute of Population Studies (NIPS), the district's estimated population was 1,003,031 in 2017, having a population density of 580 residents per square kilometer. The dominant caste or tribe of District is Awan followed by Gujjar and Tanoli. The Haripur is largely a rural district, and about only 12% of the population resides in urban areas. The temperature in the area ranges from almost 39 °C in summer to less than 10 °C in winter. Agriculture is the primary source of livelihood of the rural population of the study area. The area's economic growth depends on pastures, crop diversity, cultivation of fodder species and the development of medicinal plants and livestock diversity.

### Field survey and data collection

The entire study area was regularly and seasonally (spring, summer, winter and autumn) visited from January 2015 to January 2017. In the study area, the primary target sites were Muslimabad, Barkot, Jatti Pind, Tofkian, Khanpur, Kalinjar, Barella, Hattar, Qazipur, Ghazi, Najafpur, Jabri, Nara Amazai, Rehana, Teer, Syria, Sirikot, Bagra, Beer and Dingi. The field survey aimed to gather field data and activities, such as (1) plant’s collection, (2) local knowledge concerning plants, (3) identifying associated consequences to plants through personal observation and interviews, (4) photography and (5) medicinal plant uses along with recipes, through semi-structured questionnaires, interviews, keen observations and group discussions. The questionnaire and interview method helped to document indigenous folk knowledge by involving knowledgeable persons, traditional healers (Hakims) and local people (Table 1). Respondents were chosen by random selection of residents who were considerably connected to plants and were interested in traditional healthcare. Interviews were conducted mostly in fields, and photographs were shown for identification with local plant name. Women were interviewed indirectly through male family members. Participants were briefed about the research objectives and were allowed to discontinue the interview at any time. Each informant was interviewed regularly every season. The national language of Pakistan (Urdu) and the native language of the study area (Hindko) were used as a medium of communication. Thereafter, an English language questionnaire was filled for each informant (Additional file 1).

**Table 1:** Demographic data about informants of the study area

Variable	Demographic categories	Numbers	Percentage
Gender	Male	70	87.5
	Women	10	12.5
Experience	Traditional healer	5	6
	Herdsmen	17	21
	Farmer	52	65
	Local people	6	8
Age groups	20–40	15	19
	41–60	40	50
	Above 60	25	31
Education	Illiterate	21	26
	Primary	21	26
	Middle	16	20
	Matric and above	22	28

### Plant identification

Collected plant species were identified with the help of Flora of Pakistan, Flora of West Pakistan [28] and Flora of Punjab [29], and online Flora (www.efloras.org/). Plants names were also identified through literature, plant list (www.theplantlist.org), Medicinal plant names services (https://mpns.science.kew.org/mpns-portal/) [30]. The system proposed by Raunkiær [31, 32], and modified by Brown [33], was followed to categorize the collected plant specimens into their habits and life forms. Plants were submitted to the Herbarium, Department of Botany, Hazara University Mansehra (Pakistan), and vouchers were issued. For voucher specimen, standard herbarium techniques [34, 35] were strictly followed.

### Quantitative and correlative analysis of ethnomedicinal data

The collected ethnomedicinal data were analyzed using different quantitative analyses, including relative frequency citation (RFC), use value (UV) of medicinal plant and Jaccard index (JI) analysis by comparing the present study with published work to access knowledge variation among different communities. The obtained data were presented in percentages and proportions.

#### Relative frequency citation (RFC)

The RFC was calculated without taking into account the use categories by following the formula [36].

$${\text{RFC}} = \frac{{{\text{FC}}}}{N} \left( {0 > {\text{FRC}} > 1} \right)$$

RFC shows the importance of each species in the study area given by the FC (FC is the number of local informants reported the uses of plant species) divided by the total number of informants (*N*).

#### Use value (UV) of plant species

Use value (UV) determines the relative importance of plant species uses. It was calculated using the following formula [37].

$${\text{UV}} = \sum {Ui/N}$$

where “UV” indicates the use value of individual species, “Ui” is the number of uses recoded for a given species by each informant and “*N*” represents the number of total informants.

#### Pearson’s correlation

Pearson’s correlation, SPSS (ver. 16), tested correlation analysis between the RFC and UV.

#### Jaccard index (JI)

To compare the study with published literature and to access the similarity and dissimilarity of traditional knowledge among different communities and areas, the Jaccard index was calculated using the following formula [38].

$${\text{JI}} = \frac{c \times 100}{{\left( {a + b} \right){-}c}}$$

where ‘*a*’ represents the total number of species in area *A* (our study area), ‘*b*’ represents the number of species from other published area *B* and ‘*c*’ represents the number of common species in both A and B.

## Results

### Description of medicinal plant families

**Fig. 2:**
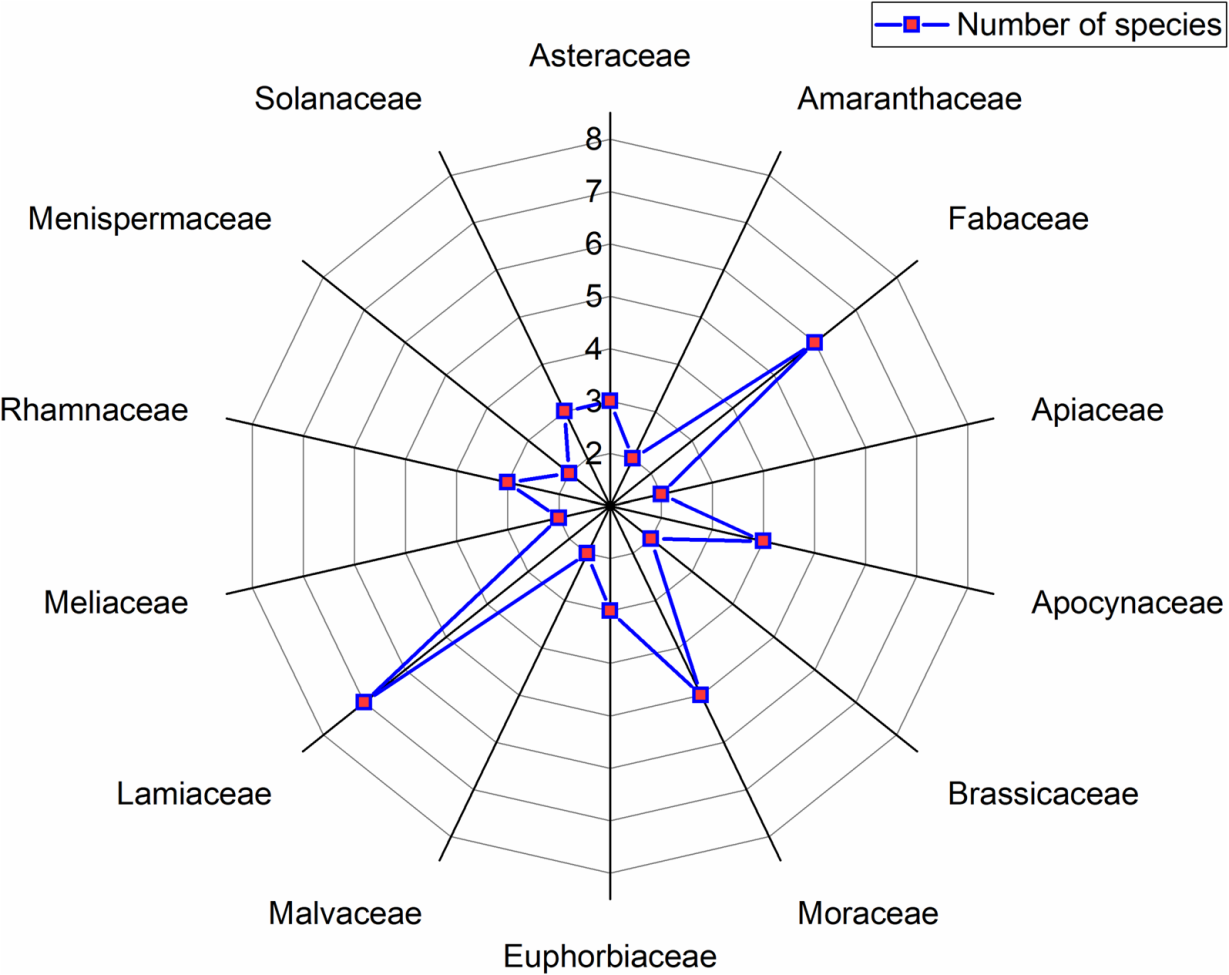
Distribution of medicinal plant species according to their family in the study area

The high diversity of plant families in the study area can be deduced from the presence of 50 different families. Among them, Lamiaceae was the largest family having 7 species, followed by Fabaceae (6 species), Moraceae (5 species), Apocynaceae (4 species), Asteraceae, Euphorbiaceae, Rhamnaceae and Solanaceae (3 species each), Amaranthaceae, Apiaceae, Brassicaceae, Malvaceae, Meliaceae, Menispermaceae (2 species each) (Fig. 2) and remaining families with one species each.

### Medicinal plant enumerations

**Fig. 3:**
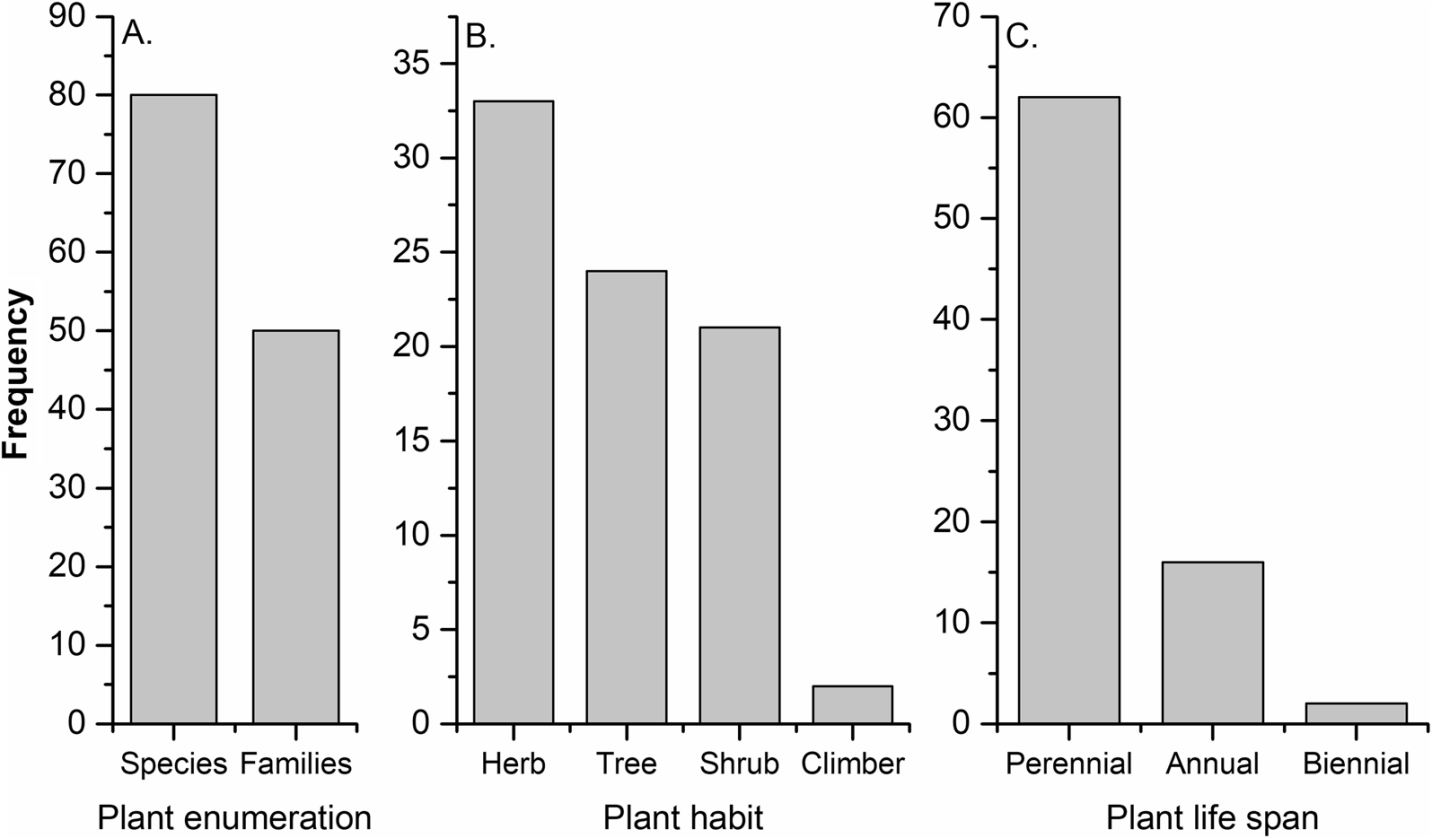
Description of medicinal **A** plant enumeration, **B** plant habit and **C** plant life span of the study area

Eighty plants were recorded covered in this study; herbs (33, 41.2%) were dominant, followed by shrubs (21, 26.2%), trees (24, 30%) and climbers (2, 2.5%). Furthermore, life spans for the majority of plants were recorded as perennial (62, 77.5%), followed by annual (16, 20%) and biennial (2, 2.5%) (Fig. 3). Among these, 40 plant species were used for livestock healthcare, and 49 plant species were used to treat human diseases, including 9 plant species which were commonly used for both (human and livestock). Complete information about each plant species includes botanical name, family, local name, voucher number, habit, life span, locality, part used, either utilized to treat human or animal diseases or both, and their recipes are listed with RFC and UV in Tables 2, 3 and 4.

**Table 2:** Ethnomedicinal plant used to treat human diseases in District Haripur, Khyber Pakhtunkhwa, Pakistan

S. no	Taxonomic name/ family, voucher no	Local name	Locality	Life habit/life span		Part used	Diseases treated	Ethnomedicinal recipes	Quantitative indices			
									FC	RFC	∑Ui	UV
1	*Achyranthus aspera* L. (Amaranthaceae), 04-Z	Puth-Kanda, Lehndi Booti	Choi	H	P	RT	Tonsillitis	External application of fresh root paste for one week twice a day	12	0.15	70	0.87
2	*Ailanthus altissima* (Mill.) Swingle (Simaroubaceae), 06-Z	Darawa	Dartian	T	P	BA	Dysentery and Diarrhea	Bark Juice (½ cup) mixed with milk and taken	13	0.16	64	0.8
3	*Allium sativum* L. (Alliaceae), 07-Z	Thoom	Khanpur	H	B	BB	High blood pressure	Two bulbs are eaten with a meal	15	0.18	42	0.52
4	*Althaea officinalis* L. (Malvaceae), 10-Z	Khatmi	Jabri	H	A	LE	Cough and tonsillitis	One cup of seed or leaf tea is used thrice a day	23	0.28	45	0.56
5	*Artemisia vulgaris* L. (Asteraceae), 12-Z	Afsanteen	Joulian	H	P	TW	Hepatitis	10-g twigs powdered with water are taken thrice times a day	22	0.27	25	0.31
6	*Azadirachta indica* A. Juss. (Meliaceae), 13-Z	Nim	Pakshai	T	P	FR	Diabetes	Powder (1 spoon) form of fruit with water, orally everyday	13	0.16	35	0.43
7	*Bauhinia variegata* L. (Fabaceae; subfamily Caesalpinioidea), 14-Z	Kalyarh, Kachnar	Garam thoon	T	P	FL	Stomach Tonic	Young flowers are cooked as a vegetable and eaten	14	0.17	62	0.77
8	*Boerhavia diffusa* L. (Nyctaginaceae), 17-Z	It-sit	Barkot	H	P	LE	Diabetes and jaundice	Decoction of leaves is taken	16	0.2	17	0.21
9	*Caralluma edulis* (Edgew.) Benth. ex Hook.f. (Apocynaceae), 23-Z	Chong	Karwali	H	P	ST	Diabetes	One cup of stem juice is taken thrice times a day	29	0.36	65	0.81
10	*Catharanthus roseus* (L.) G.Don *(*Apocynaceae)*,* 29-Z	Sadabahar	Bagra	H	p	LE	Wasp-sting	Leaf juice is applied	12	0.15	66	0.82
11	*Celtis australis* auct. non L. (Ulmaceae), 30-Z	Batkhar	Najafpur	T	P	FR	Stomach problems	10 g of fruit powdered is taken with water	13	0.16	24	0.3
12	*Cichorium intybus* L. (Asteraceae), 37-Z	Kasni	Kotla	H	P	RT	Stomach problem	Grinded root is taken	12	0.15	15	0.18
13	*Cissampelos pareira* L. (Menispermaceae), 42-Z	Phalaan jarhi, Ghora-sum	Dhuniya	C	P	LE	Wounds	Leaves are crushed and applied	14	0.17	36	0.45
14	*Colebrookea oppositifolia* Sm. (Lamiaceae), 44-Z	Shakardana	Dhuniya	S	P	LE	Cough	Leaves are chewed	13	0.16	34	0.42
15	*Datura stramonium* L. (Solanaceae), 58-Z	Datura	Dara	H	A	FL, LE	Bleeding piles	Powdered flowers and leaves are used as an ointment	12	0.15	35	0.43
16	*Diospyros lotus* L. (Ebenaceae), 60-Z	Amlok	Shah kabul	T	P	FR	Chest phlegm	Fruit is eaten	16	0.2	19	0.23
17	*Eucalyptus globulus* Labill. (Myrtaceae), 67-Z	Gond	Khanpur	T	P	RE	Cuts and wounds	Resin is applied externally	13	0.16	25	0.31
18	*Ficus carica* L. (Moraceae), 78-Z	Anjeer	Ranjha	T	P	FR	Blood deficiency	Fruit is eaten	25	0.31	32	0.4
19	*Ficus palmata* Forssk*.* (Moraceae), 79-Z	Phagwari	Ranjha	T	P	FR	Blood deficiency and abdominal problems	The fruit is left in the water overnight and eaten as a first food in the morning	13	0.16	45	0.56
20	*Malva sylvestris* L. (Malvaceae), 120-Z	Khabazi	Kohala	H	B	LE	Chest infection and asthma	One cup of leaf tea is taken 2–3 times a day	13	0.16	32	0.4
21	*Mentha longifolia* L. (Lamiaceae), 131-Z	Chita podna	Bhamala	H	P	LE	Fever, dysentery and vomiting	Leaf tea is used	13	0.16	24	0.3
22	*Myrsine africana* L. (Primulaceae), 148-Z	khokonr	Najafpur	S	P	FR	Anthelmintic	Fruit is eaten	13	0.16	35	0.43
23	*Nasturtium officinale*W.T.Aiton (Brassicaceae), 155-Z	Tara meera	Chaskalawaan	H	P	LE	Constipation, diuretic and obesity	Cooked vegetable of leaves is eaten	6	0.07	62	0.77
24	*Ocimum basilicum* L. (Lamiaceae), 174-Z	Niaz-bo	Desra	H	A	LE	Skin care	Leaf juice is applied	16	0.2	32	0.4
25	*Olea ferruginea* Royle (Oleaceae), 186-Z	Kaho	Garam thoon	T	P	LE	Skin pimples	Leaf tea is used	23	0.28	45	0.56
27	*Oxalis corniculata* L. (Oxalidaceae), 197-Z	Khat-matra	Halli	H	A	LE	Skin inflammations	Powdered leaves are applied as a poultice	12	0.15	34	0.42
28	*Papaver somniferum* L. (Papaveraceae), *202-Z*	Khashkhash	Halli	H	A	FR	Chest infection and cough	Tea of dried fruit is taken	11	0.13	45	0.56
29	*Pinus roxburghii* Sarg. (Pinaceae), 212-Z	Chir	Bagla	T	P	RE	Skin problems	Resin is applied externally	13	0.16	25	0.31
30	*Pistacia chinensis* subsp. integerrima (J.L.Stewart) Rech.f. (Anacardiaceae), 218-Z	Kangur	Chasklawaan	T	P	BA	Jaundice	½ cup of bark decoction is taken daily	13	0.16	39	0.48
31	*Rubus fruticosus* L. (Rosaceae), 235-Z	Garacha	Ranjha	S	P	FR	Carminative	Fruit is eaten	15	0.18	42	0.52
26	*Rydingia limbata* (Benth.) Scheen & V.A. Albert (Lamiaceae), 192-Z	Chita Kanda, Bamboli	Old Khanpur	S	P	WP	Wounds	The powder of whole plant mixed with butter before being applied	24	0.3	36	0.45
32	*Sageretia thea* (Osbeck) M.C.Johnst. (Rhamnaceae), 252-Z	Gangeeri	Rajdhani	S	P	FR	Diabetes and kidney stones	Dried powdered (½ spoon) of fruit, taken in the morning and evening with water	10	0.12	42	0.52
33	*Sisymbrium irio* L. (Brassicaceae), *257-Z*	Khub kalan	Kot-jandaan	H	A	LE	Chest infection	Leaves infusion is given	8	0.1	26	0.32
34	*Solanum nigrum* L. (Solanaceae), 260-Z	Kach mach	Najafpur	H	A	LE	Asthma	Tea of shade dried leaves is taken	16	0.2	43	0.53
35	*Tamarindus indica* L. (Fabaceae), 268-Z	Imli	Bees ban	T	P	PP	Fever and liver tonic	Juice of pulp is drunk daily	23	0.28	50	0.62
36	*Tribulus terrestris* L. (Zygophyllaceae), 274-Z	Gokhru	Neelan bhoto	H	A	LE	Male sexual weakness	A few leaves are soaked for a while in a glass of water and taken three times daily	13	0.16	33	0.41
37	*Viola odorata* L. (Violaceae), 277-Z	Ba-nafsha	Kharian	H	A	LE	Cough, cold and flu	Leaf tea is taken	27	0.33	73	0.91
38	*Woodfordia fruticosa* (L.) Kurz (Lythraceae), 279-Z	Taawi, Dhawi	Shah Kabul	S		LE	Skin diseases	A poultice of leaves is applied externally	9	0.11	45	0.56
39	*Zanthoxylum armatum* DC. (Rutaceae)*,* 280-Z	Timber	Halli	S/T	P	SD	Jaundice	One spoon of dried or fresh seeds powdered is taken daily	32	0.4	60	0.75
40	*Ziziphus nummularia* (Burm.f.) Wight & Arn. (Rhamnaceae)*,* 281-Z	Beri	Sarhadna	S	P	LE	Wounds	Paste of grinded leaves are applied	13	0.16	38	0.47

**Table 3:** Ethnomedicinal plant used to treat livestock diseases in District Haripur, Khyber Pakhtunkhwa, Pakistan

S. no	Taxonomic name/ family, voucher no	Local name	Locality	Life habit/life span		Part used	Animal treated	Animal disease treated	Ethnoveterinary recipes	Quantitative indices			
										FC	RFC	∑Ui	UV
1	*Allium cepa* L. (Amaryllidaceae), 08-Z	Payaz	Khanpur	H	P	BB	Goat, buffalo and cow	Mouth infections	Grinded bulb mixed with black salt is given with water	13	0.16	35	0.43
2	*Amaranthus viridis* L. (Amaranthaceae), 11-Z	Chaleray	Jabri	H	A	LE	Buffalo and cow	Milk Production	A decoction of leaves is given with a small amount of salt	13	0.16	65	0.81
3	*Berberis lycium* Royle (Berberidaceae), 16-Z	Simbulu	Dartian	S	P	RT	Goat, buffalo and cow	Wounds and internal injury	The powdered root bark is applied to wounds. It is also given for internal injury	35	0.43	71	0.88
4	*Bombax ceiba* L. (Bombacaceae), 19-Z	Sambal	Daboola	T	P	ST, BA	Goat, buffalo and cow	Dislocated bones	Paste of stem bark mixed with turmeric (haldi) and applied	11	0.13	39	0.48
5	*Calotropis procera* W. T. Aiton (Asclepiadaceae), 20-Z	Akk	Ghazi	S	P	LA	Cow, buffalo and goat	Wounds	Latex is applied externally	13	0.16	81	1.01
6	*Cannabis sativa* L. (Cannabaceae), 22-Z	Pang, bhang	Hattar	S	A	LE	Cow, buffalo and goat	Loss of appetite	Fresh leaves are fed	12	0.15	45	0.56
7	*Carissa opaca* Stapf ex Haines (Apocynaceae)*,* 26-Z	Garinda	Choi	S	P	LE	Cow, buffalo and goat	Foot and mouth disease	Leaves are crushed and fed	31	0.38	25	0.31
8	*Cassia fistula* L. (Fabaceae; subfamily Caesalpinioidea), 28-Z	Kinjal, Amaltas	Ranjha	T	P	PD	Cow, buffalo and goat	Asthma and pneumonia	Dried pod powder is given orally	30	0.37	45	0.56
9	*Chenopodium album* L. (Chenopodiaceae), 34-Z	Bthawa	Kohala	H	A	WP	Goat and cow	Wound healing	The paste is applied to wounds	13	0.16	68	0.85
10	*Coriandrum sativum* L. (Apiaceae), 46-Z	Dhania	Beer	H	A	LE, RT	Buffaloes	Antidiuretic	Root and leaves decoction is given for 5 days	13	0.16	19	0.23
11	*Curcuma longa* L. (Wild) (Zingiberaceae), 49-Z	Haldi	Khanpur	H	P	LE	Cow and goat	Wound healing	A decoction of leaves is given for 3 days	37	0.46	79	0.98
12	*Cynodon dactylon* (L.) Pers. (Poaceae), 54-Z	Khabal	Nara Amazai	H	P	WP	Buffaloes, cow and goat	Hematuria	Plant juice is given twice a day for a week	41	0.51	65	0.81
13	*Dalbergia sissoo* Roxb. ex DC. (Fabaceae), 55-Z	Taali, Sheesham	Bareela	T	P	LE	Cow, buffalo and goat	Diarrhea	Leaf paste with a little amount of salt is given	12	0.15	25	0.31
14	*Dodonaea viscosa* (L.) Jacq. (Sapindaceae), 65-Z	Sanatha	Garam Thoon	S	P	LE	Cow, buffalo and goat	Bone fracture	Leaves are heated and mixed with soil, then tied over the fracture	62	0.77	34	0.42
15	*Euphorbia helioscopia* L. (Euphorbiaceae), 70-Z	Chhatri Dodak	KotnajibUllah	H	A	LE and SD	Goat, buffalo and cow	Food poisoning	Powdered leaves and seeds are given with water	12	0.15	19	0.23
16	*Ficus benghalensis* L. (Moraceae), 76-Z	Bohr	Bandi	T	P	RT	Goat, buffalo and cow	Diarrhea and dysentery	A paste of prop root along with honey is given	18	0.22	55	0.68
17	*Grewia optiva* J.R. Drumm. ex Burret (Tiliaceae), 90-Z	Dhaman	Babootri	T	P	LE	Buffalo	Easy delivery	Leaves are fed	57	0.71	85	1.06
18	*Lantana camara* L. (Verbenaceae), 102-Z	Chandni	Hattar	S	P	LE and TW	Goat, buffalo and cow	Joint pains	Decoction is given	10	0.12	29	0.36
19	*Mallotus philippensis* (Lam.) Müll. Arg. (Euphorbiaceae)*,* 114-Z	Kamila	Noorpur	S	P	FR	Goat, buffalo and cow	Intestinal worms	Dried powdered fruit is given for 3 days	56	0.7	52	0.65
20	*Mentha arvensis* L. (Lamiaceae), 130-Z	Podina	Bhamala	H	P	LE	Cow, buffalo and goat	Dysentery	Fresh leaves along with black salt are given	52	0.65	36	0.45
21	*Morus alba* (L.) Roxb. (Moraceae), 144-Z	Chita toot	Dara	T	P	FR and LE	Goat, cow and buffalo	Mastitis	Decoction is given	23	0.28	35	0.43
22	*Nerium oleander* L. (Apocynaceae), 162-Z	Kundair	Najafpur	S	P	WP	Goat	Stomachache	The dried powdered plant is given with water in a small quantity	8	0.1	55	0.68
23	*Punica granatum* L. (Punicaceae), 221-Z	Daruna	Barkot	S	P	LE and FR	Goat, cow and buffalos	Anthelmintic	Decoction is given	45	0.56	83	1.03
24	*Ricinus communis* L. (Euphorbiaceae), 230-Z	Arand	Mang	Sb	P	SD	Cow, buffalo and goat	Constipation	Seed oil is given along with fodder	16	0.2	40	0.5
25	*Salvia moorcroftiana* Wall. ex Benth (Lamiaceae), 254-Z	Kallijari	Kohala	H	P	RT	Goat, buffalo and cow	Internal injuries	Decoction is given	11	0.13	46	0.57
26	*Solanum surattense* Burm. f. (Solanaceae), 264-Z	Mohree	Khoi Kaman	H	A	WP	Goat, buffalo and cow	Fever	Crushed plant mixed with flour is given	17	0.21	64	0.8
27	*Taraxacum officinale* F.H. Wigg. (Asteraceae), 270-Z	Hand	Dara	H	P	WP	Goat, buffalo and cow	Milk deficiency	The whole plant is fed	6	0.07	9	0.11
28	*Tinospora cordifolia* (Willd.) Miers. (Menispermaceae), 271-Z	Gulo	Kotla	C	P	ST and LE	Goat, buffalo and cow	Fever	decoction form is used continuously for 4 days	9	0.11	13	0.16
29	*Trichodesma indicum* (L.) R. Br. (Boraginaceae), 275-Z	Kali booti	Halli	H	A	LE	Cow, buffalo and goat	Inflammation and swellings	Leaves poultice is applied externally	11	0.13	28	0.35
30	*Vitex negundo* L. (Lamiaceae), 278-Z	Somali, MarvanI	Choi	S	P	LE	Cow, buffalo and goat	Fractured bones	Warmed leaves are tied over the fractured bones	13	0.16	38	0.47
31	*Ziziphus jujuba* Mill*.* (Rhamnaceae), 283-Z	Bairi	Karwali	T	P	LE	Cow, buffalo and goat	Dysentery	Decoction is given	19	0.23	41	0.51

**Table 4:** Ethnomedicinal plant used to treat human and livestock diseases in District Haripur, Khyber Pakhtunkhwa, Pakistan

S. no	Taxonomic name/family, voucher no	Local name	Locality	Life habit/life span^a^		Part used^b^	Organism treated	Disease treated	Ethnomedicinal recipes	Quantitative indices^c^			
										FC	RFC	∑Ui	UV
1	*Acacia modesta* Wall (Fabaceae; subfamily Mimosoideae), 03-Z	Phulai	Dartian	T	P	LE, SD	Cow and buffalo	Delivery	A decoction of leaves and seeds is given for 3 days	16	0.2	55	0.68
						GM	Human	Backache	Gum is fried with wheat flour in "desi ghee." This is known as "Halwa" in the local community and given especially to women after delivery				
2	*Acacia nilotica* (L.) Delile M. (Fabaceae; subfamily Mimosoideae), 02-Z	Kikar	Najafpur	T	P	SP,	Cow and buffalo	Colic pain	A decoction of spines is given for 3 days	13	0.16	40	0.5
						GM	Human	Stomach ulcer	Powdered gum at 10-g with milk/water is taken				
3	*Adhatoda vasica* Nees. (Acanthaceae), 05-Z	Bhaikur, Aroosa	Halli	S	P	LE	Cow, buffalo and goat	Wounds and inflammations	externally application of leaves poultice	17	0.21	75	0.93
						LE	Human	Diabetes and jaundice	One cup of leaves juice is taken in a day				
4	*Aloe barbadensis* Mill. (Liliaceae), 09-Z	Kanwar-ghandal	Dara	H	P	RT	Cow, buffalo and goat	Gastro-intestinal	Powdered roots are given with water for 4 days	14	0.17	32	0.4
						LE	Human	Skin problems	Leaf gel is burnt over the frypan and applied externally				
5	*Centella asiatica* (L.) Urb. (Apiaceae), 33-Z	Barhami	Neelan bhoto	H	P	LE	Goat, buffalo and cow	Diarrhea	Leaves are roasted and cooled, fed twice a day for 3 days	37	0.46	37	0.46
						LE	Human	Throat problems	Leaf tea is used				
6	*Melia azedarach* (L.) Pers (Meliaceae), 124-Z	Daraik, Bakain	Sarhadna	T	P	LE		Intestinal worm and stomach flatulence	Crushed leaves are fed along with bamboo leaves	65	0.81	70	0.87
						LE	Human	High blood pressure	1 spoon of powdered fruit is taken at night with water				
7	*Morus nigra* L. (Moraceae), 145-Z	Kala toot, She-toot	Dara	T	P	LE	Buffalo and cow	Laxative	Dried powdered leaves are given	17	0.21	25	0.31
						FR	Human	Cough and throat infection	Ripped fruit is eaten				
8	*Phyllanthus emblica* L. (Phyllanthaceae), 208-Z	Amla	Daboola	T	P	FR	Buffalo cow and goat	Anthrax	Powdered fruit with *Ocimum basilicum* leaves is given orally	15	0.18	66	0.82
						FR	Human	Fever	The fruit is cooked in “lassi” and eaten				
9	*Rumex hastatus* D.Don (Polygonaceae), 238-Z	Katmal, Tehtur	Najafpur	S	P	WP	Cow, buffalo and goat	Appetite	The whole plant is fed	9	0.11	31	0.38
						WP	Human	Diarrhea	Powdered roots are given with water				

^a^Life Habit/Life span; S, Shrubs; H, Herbs; C, Climbers; T, Trees; A, Annual; B, Biennial; P, Perennial

^b^Plant Part(s); RT, Root; LE, Leaf; ST, Stem; FR, Fruit; SH, Shoot; WP, Whole Plant; BA, Bark; SD, Seed; RH, Rhizome; FL, Flower; GM, Gum; RE, Resin; BB, Bulb; TW, Twigs; PP, Pulp; SP, Spine

^c^Quantitative Indices; RFC = Relative frequency of citation, FC = Frequency citation, ∑Ui = sum of uses, UV = Use values

### Plant part(s) used

**Fig. 4:**
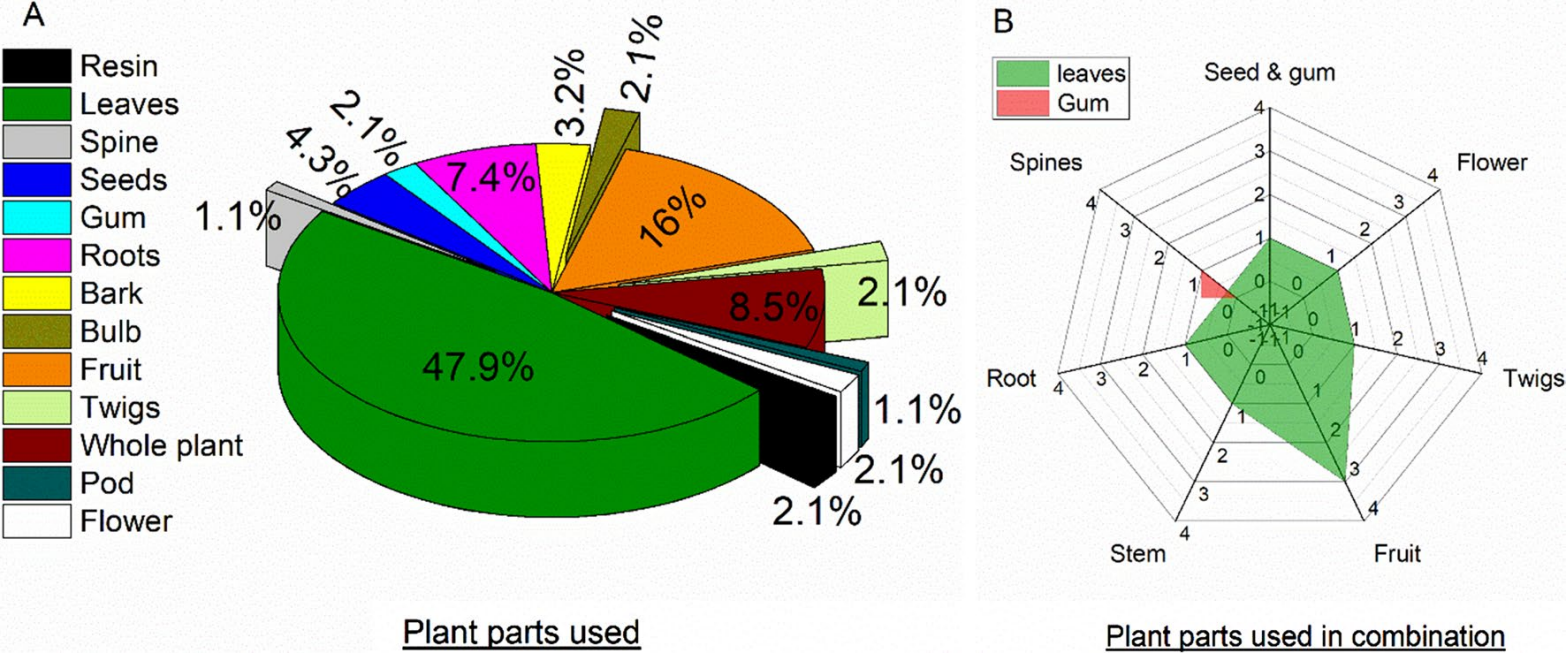
Utilization of **A** plant parts **B** plant parts in combination for management of livestock and human diseases

Locals utilized different plant parts (either in combination or separately) in the study area for the management of livestock and human diseases. Among them, leaves (47.9%) were the most commonly used part in herbal preparations, followed by fruits (16%), whole plant (8.5%), roots (7.4%), seeds (4.3%), bark (3.2%), gum, bulb, twigs, flower, resin (2.1% each), spines and pods (1.1% each) (Fig. 4A). In combination, leaves were the most common plant parts combined/utilized with fruits (3), flower, roots, seed and gum, twigs and stem (1 each). The combination of gum with the spine was utilized only one time (Fig. 4B).

### Mode of preparation, administration and application

**Fig. 5:**
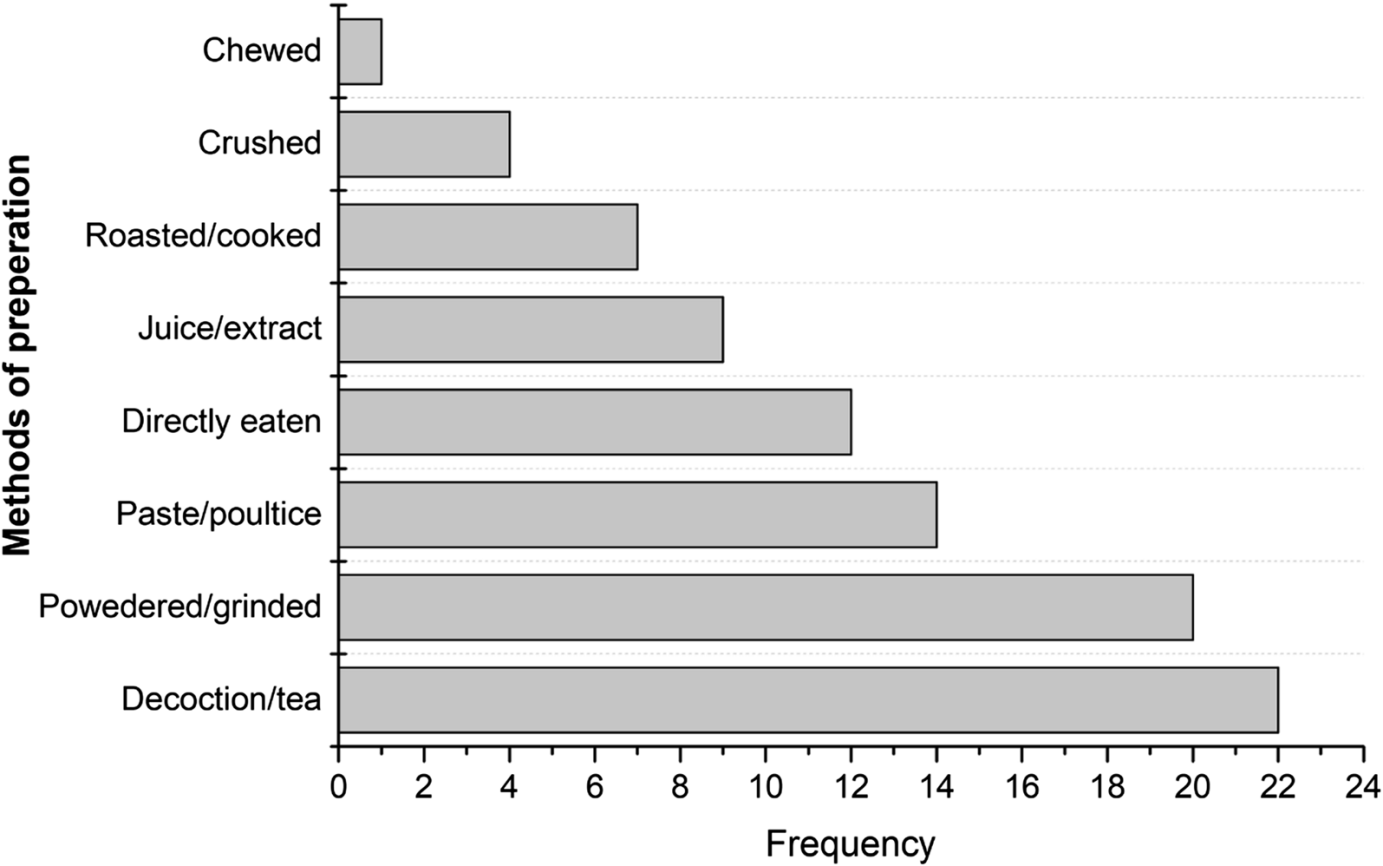
Commonly used methods in the preparation of plant recipes

The remedies/recipes preparations of the 80 plant species are categorized according to their type of preparation, which revealed that decoction/tea (22 species) was a widely used preparation method by locals, followed by powdered/grinded (20 species), paste/poultice (14 species), directly eaten (12 species), juice/extract (09 species), roasted/cooked (07 species), crushed (04 species) and chewed (one species) (Fig. 5). It was also recorded that the local people use preparations/recipes of ethnomedicinal plant, both as externally (25%) and internally (75%) application.

### Species richness for the management of human and livestock diseases

Local people of the study area used 49 medicinal plants to treat 42 different ailments related to humans. These 42 ailments were further categorized into 12 major diseases categories. It was found that single medicinal plant species can treat several human ailments, and several medicinal plant species can treat single disease. In the study area, 34 livestock ailments were identified to be treated by 40 medicinal plants. These 34 ailments were further categorized into 07 major disease categories (Table 5). The ethnoveterinary medicinal plants were utilized mostly for cows (35%), followed by buffaloes (34%) and goats (31%).

**Table 5:** Major disease categories of human and livestock with number of remedies

Disease treated	No. of remedies	
	Human ailments	Livestock ailments
Digestive system-related	12	16
Dermal and wound problems	10	05
Liver tonic and jaundice	06	–
Diabetes	05	–
Respiratory system-related	05	02
Circulatory system and blood-related	04	–
Mouth and throat infections	03	02
Excretory system-related	02	02
Fever	02	02
Animal and insect bites	01	–
Bones and joints related	01	06
Reproductive system-related	01	05

### Quantitative analysis

To analyze ethnomedicinal data, quantitative value indices were determined in this study. The RFC value ranges from 0.07 to 0.81 for the recorded species, and the highest value of RFC was recorded for *Melia azedarach*, *Dodonaea viscosa*, *Grewia optiva* and *Mallotus philippensis* (0.81, 0.77, 0.71 and 0.7), respectively.

The UV of plant species determines the relative importance of plants in the study area. The UV values for *Curcuma longa, Adhatoda vasica, Viola odorata, Berberis lycium, Achyranthus aspera, Melia azedarach and Chenopodium album* were 1.06, 1.01, 1.03, 0.98, 0.93, 0.91, 0.88, 0.87, 0.87 and 0.85, respectively, *Phyllanthus emblica* and *Catharanthus roseus* (0.82 each), *Amaranthus viridis, Cannabis sativa,* and *Cynodon dactylon* (0.81 each), and *Ailanthus altissima* and *Solanum surattense* (0.8 each). The other remaining plant species were recorded with a UV value of < 0.81, which indicated that they were less exploited by local people (Table 2). RFC and UV were significantly correlated (Pearson’s test; *p* = 0.01), and the correlated values explained approximately 31% of the data (Additional file 2: Table S1).

### Comparison and Jaccard index

The comparative analysis exhibited a significant difference in the medicinal plant utilization among different communities of Pakistan. Twenty national studies from different areas of Pakistan were compared with the present study. Overall, 49 species were reportedly used to manage human diseases. Similarity percentages ranged from 0 to 57.1%. The similarity index (JI) value ranges from 1.76 to 16.85 (Table 6).

**Table 6:** Comparative analysis between this study and other studies from Pakistan of medicinal plant used for human diseases management

Area	Study year	Number of recorded plant species	Plants with similar use	Plants with dis-similar use	Total species common in both area	Species enlisted only in aligned areas	Species enlisted only in study area	% of plants with a similar use	% of plants with dis-similar use	JI	Citation
Chitral	2017	36	01	05	06	30	74	16.6	83.3	6.12	[39]
District Charsadda	2016	60	04	03	07	53	73	57.1	42.8	5.88	[40]
Indus river	2014	70	04	07	11	59	69	36.3	63.6	9.40	[13]
District Abbottabad	2013	67	03	08	11	56	69	27.2	72.7	9.64	[41]
Hingol national park	2012	39	0	03	03	36	77	0	100	2.72	[42]
Neelum valley (AJK)	2012	39	0	02	02	37	78	0	100	1.76	[43]
Khushab	2012	14	0	04	04	10	76	0	100	4.87	[44]
District Attock	2011	43	01	03	04	39	76	25	75	3.60	[45]
District Sialkot	2011	48	02	04	06	42	74	33.3	66.6	5.45	[46]
Kalat and Khuzdar	2010	61	01	02	03	58	77	33.3	66.6	2.27	[47]
District Bannu	2010	27	01	04	5	22	75	20	80	5.43	[48]
District Abbottabad	2010	54	05	10	15	39	65	33.3	66.6	16.85	[49]
Northern Pakistan	2009	27	0	02	02	25	78	0	100	1.98	[50]
Tehsil Chakwal	2009	29	01	03	04	25	76	25	75	4.12	[51]
Gilgit	2008	98	02	06	08	90	72	25	75	5.19	[52]
District Mianwali	2007	21	01	01	02	19	78	50	50	2.10	[53]
Bagh (AK)	2007	33	0	3	3	30	77	0	100	2.88	[54]
Mahal (Kohistan)	2007	50	02	02	4	46	76	50	50	3.38	[55]
M2 motorway	2007	81	04	09	13	68	67	30.7	69.2	10.65	[56]
Siran valley	2006	80	01	13	14	66	66	7.1	92.8	11.86	[57]

Furthermore, 25 national studies from the different areas of Pakistan were compared with the present 40 reported species for management of veterinary diseases. The similarity percentage ranges from 0 to 60%. The degree of similarity index (JI) value ranges from 1.17 to 32.78 (Table 7).

**Table 7:** Comparative analysis between this study and other studies from Pakistan of medicinal plant used for the management of livestock diseases

Area	Study year	Total species recorded	Plants with similar uses	Plants with dis-similar uses	Total species common in both area	Species enlisted only in aligned areas	Species enlisted only in study area	% of plants with a similar use	% of plants with dis-similar use	JI	Citation
FATA, Pakistan	2018	94	02	14	16	78	64	12.5	87.5	12.69	[58]
Bajaur Agency, Pakistan	2018	73	02	13	15	58	65	13.3	86.6	13.88	[59]
District Jhang, Pakistan	2017	46	01	11	12	34	68	8.3	91.6	13.33	[60]
Neelum Valley, Pakistan	2017	50	00	04	04	46	76	00	100	3.38	[61]
Chail valley, Pakistan	2017	55	02	05	07	48	73	28.5	71.4	6.14	[62]
Hangu, Pakistan	2016	24	01	07	08	16	72	12.5	87.5	10	[63]
Karak, Pakistan	2015	46	02	09	11	35	69	18.1	81.8	11.82	[64]
Peshawar, KPK, Pakistan	2015	83	02	09	11	72	69	18.1	81.8	8.46	[65]
Sulaiman Range, Pakistan	2014	41	12	08	20	21	60	60	40	32.78	[66]
DI Khan, Pakistan	2014	43	01	07	08	35	72	12.5	87.5	8.08	[67]
Tharparkar, Pakistan	2014	22	00	02	02	20	78	00	100	2.08	[68]
Malakand valley, Pakistan	2014	28	04	05	09	19	71	44.4	55.5	11.11	[16]
Lesser Himalaya, Pakistan	2013	89	06	13	19	70	61	31.5	68.4	16.9	[69]
Baffa, Pakistan	2012	30	08	06	14	16	66	57.1	42.8	20.5	[70]
Allai, Pakistan	2012	24	00	03	03	21	77	00	100	3.15	[71]
Jhang, Pakistan	2012	35	00	05	05	30	75	00	100	5	[72]
Northern Pakistan	2012	54	03	08	11	43	69	27.2	72.7	10.8	[19]
Poonch Valley, Azad Kashmir	2012	19	04	03	07	12	73	57.1	42.8	8.97	[73]
Hilly area, Pakistan	2010	35	01	04	05	30	75	20	80	5	[74]
Suleiman region, Pakistan	2010	08	0	1	01	7	79	00	100	1.17	[75]
Sargodha, Pakistan	2009	25	00	01	01	24	79	00	100	0.98	[27]
Cholistan dessert, Pakistan	2009	35	01	02	03	32	77	33.3	66.6	2.83	[76]
Faisalabad, Pakistan	2009	39	01	05	06	33	74	16.6	83.3	5.94	[77]
Kashmir Himalaya, Pakistan	2007	24	00	02	02	22	78	00	100	2.04	[78]
Samahni valley, Pakistan	2006	54	03	12	15	39	65	20	80	16.85	[79]

### Major threats to plant diversity

Plant resources are under severe threats; the major threats (fires, overgrazing, overexploitation and mining activities) were observed in the visited localities of the study area. Among them, the plant diversity of Garmthun, Najafour, Dartian, Baghpur dehri and Jabri was exposed to all these major threats. Moreover, Sarae Nehmat Khan and Ghazi were less/non exposed to the threat activities except only overgrazing (Table 8).

**Table 8:** Major threats to the medicinal plant observed in District Haripur, Khyber Pakhtunkhwa, Pakistan

Locality/threat	Mining activities	Over exploitation	Over grazing	Fire
Khanpur	+	−	+	+
Beer	−	+	+	+
Garmthun	+	+	+	+
Najafpur	+	+	+	+
Hattar	−	−	+	+
Jabri	+	+	+	+
Baghpur dehri	+	+	+	+
Dartian	+	+	+	+
Nilan Bhoto	−	+	+	−
Babotri	−	+	+	+
Pharala	+	+	+	−
Ghazi	−	−	+	−
Kohala	+	−	+	+
Sarae Nehmat Khan	−	−	+	−
Nara Amazai	−	+	+	−

Data: + presence, − absence

## Discussion

The utilization of medicinal plant species belonging to the dominant plant families (Lamiaceae, Moraceae, Apocynaceae, Asteraceae, etc.) in the study area suggests that the families may have wide distribution, or the plant species are well known to communities for their medicinal purpose. The traditional knowledge of various plant families had been published around the world; among them, Asteraceae, Lamiaceae and Moraceae are well known for their medicinal purpose among the people of Pakistan [20], and other parts of the world [3, 80–82]; this knowledge may be transferred over many different communities. In the traditional medicine system, herbaceous medicinal plant have been commonly used on a large scale compared to other types of plants [83–86]. The medicinal plant or their parts are collected in different seasons depending upon their availability or frequency of active constituent deposition. The accessibility and availability of plant species may also involve their utilization rate, such as perennial plants having longer life cycles than other plant life cycles [1, 87–89]. Thus, indigenous communities in the present study area were more likely to prefer perennial plants due to their long life-cycle and availability.

Plant parts, modes of preparation and application play a significant role in herbal medicine [90]. Most herbalists believe that plant leaves have various bioactive chemical compounds which can be easily extracted [5, 91]. Leaves were the most exploited part for medicinal purposes in the present study and several other studies [92, 93]. Furthermore, the collection of leaves may not threaten the plant survival compared to the collection of the whole plant, stem, or roots, which can drive the plant species to extinction if over-collected [94]. While extraction from fresh material would be considered more useful to avoid microbial fermentation [95], previous studies demonstrated that decoction is the most commonly used preparation method for ethnobotanical medicines by traditional healers in herbal recipes [96, 97]. This method may be commonly used due to its simplicity [98], or due to the heating process which speeds up biological reaction and results in higher availability of bioactive compounds [99–101]. In our study area, other areas of Pakistan [5, 82, 102, 103] and a few other countries [104–108], the most frequently used method of plant-based medicine preparation is decoction. In regard to the various preparation methods documented in our study, other studies have also revealed similar findings; the most frequently used method of preparation in Azad Jammu and Kashmir, Pakistan was decoction (18%), followed by powder and juice (17%), paste (15.5%), chewing (11%), extract (8%), infusion (7%) and poultice (5.5%) [97].

The traditional knowledge of herbal remedies for the management of various diseases may vary due to cultural differences, areas and communities. However, it is also believed that one plant species/part can treat various types of disease due to its diverse chemical constituents. Likewise, the present study demonstrated the traditional uses of *Achyranthus aspera* roots for tonsillitis, while its leaves were previously practiced for wound healing [109], *Datura stramonium* for bleeding piles, while in Haramosh and Bugrote Valleys, Pakistan, its leaves are practiced for injuries, wounds, bleedings and pains [52], *Zanthoxylum armatum* for jaundice, while in southern Himalayan regions of Pakistan, its branches are employed for toothache and edible fruits in cardiac disorders [110]. Moreover, in comparison with other studies revealed that some species have similar uses, and some plant species are exploited for different diseases [111–113], in addition to the folk herbal medicinal literature.

Likewise, some plant species we recorded in our study area reflect similar traditional veterinary uses compared to other traditional knowledge of ethnomedicinal plant studies. For example, *Mallotus philippensis* seed powder is used in abdominal worms to remove the threadworms [73], and *Melia azedarach* is used to reduce intestinal worm load in cattle, recoded with high a (100%) fidelity level [114]. In contrast, some studies reflect dissimilar traditional uses of plants, such as *Grewia optiva* for wound healing [58], leaves paste of *Dodonaea viscosa* is used as tonic and for wound healing [62, 115], fruits of *Solanum surattense* are used for pregnancy improvement [115]*,* and in curing myiasis [31], the leaves and shoot of *Carissa opaca* are fed to increase the milk yield in goats [116], *Berberis lycium* root and stem powder for treat trauma in livestock in Afghanistan [117], *Punica granatum* is used in foot infection [118], fever, dehydration, internal parasite, tonic, indigestion, paralysis, gastric troubles, burns, flatulence and several other diseases [32, 118, 119], *Adhatoda vasica* leaves are used for cough in cow and goat [120], *Cynodon dactylon* leaves are used in burn injuries [121], leaves of *Mentha arvensis* are given to the animal if he stops taking food and also to cure bloat [116], *Curcuma longa* rhizome and *Acacia modesta* gums are used for skeleton-muscular ailments [122], and *Amaranthus viridis* fresh plant was given to cattle as purgative in case of constipation [19], Furthermore, *Acacia nilotica* is used to treat jaundice and dysentery [123], which may reflect the novel and the new uses of plant species in the present study area. Comparing present findings with previous studies shows that the same medicinal plant are used in different parts of the country for different diseases. Moreover, people also used different plant parts of the same plant for similar or different diseases.

Use value and RFC value are dynamic as it changes with area and depend on the traditional knowledge of the local people, so the UV and RFC value may vary within the same area or area to area and community to community [90]. The plant species with low UV or RFC value is considered less important species for the local people; in fact, young people may have limited knowledge to these species and may consider them unimportant, which is an alarming risk to traditional knowledge that is dependent on transference from generation to generation. As a result, this knowledge may gradually disappear.

Indigenous knowledge of the people may vary greatly due to discrepancies in their origins and cultures. Documenting and comparing this knowledge may reveal a considerable depth of knowledge among communities, resulting in novel sources for drug development [124]. Such studies also illustrate the value of indigenous medicinal plant information, with disparities between areas arising as a result of ecological [125], historical [126], phytochemical and even organoleptic differences [127]. Similar in terms of their cultural values and climatic conditions to the study area, the Jaccard index showed significant results; the highest degree of similarity index was with studies by Abbasi et al. [49], Shah and Khan [57], Ahmad [56], Mussarat et al. [13], with JI values 16.85, 11.86, 10.65 and 9.40, respectively, for the management of human diseases. Likewise, Tariq et al. [66], Abbasi et al. [69], Ch et al. [79],, Badar et al. [60] had JI values of 32.78, 16.9, 16.85, 13.88 and 13.33, respectively, for ethnoveterinary medicinal plant. The studies might have a cross-cultural exchange of knowledge between the communities through any means, historical and ecological factors, common ethnic values and similar vegetation types. The lowest JI values were for the studies conducted by Ahmad et al. [43], Afzal et al. [50], with JI values 1.76 and 1.98, respectively, for human disease management. Likewise, Dilshad et al. [27], Raziq et al. [75], Khuroo et al. [78] and Mirani et al. [68] had JI values of 0.98, 1.17, 2.04 and 2.08, respectively, for ethnoveterinary medicinal plant. These findings are in agreement with studies carried out by Kayani et al. [128]. This might be due to a greater difference in ethnobotanical knowledge due to differences in population size, species diversity, habitat structure, or less chance of exchanging cultural knowledge between the areas. The Jaccard index analysis may strengthen the value of reported medicinal plant species with their matching uses to other studies, which may provide a baseline for phytochemical, and pharmacognostic studies.

On the other hand, the JI analysis may reflect the novel uses of medicinal plant from the present study area, which may be due to the areas: (1) unique phytogeography, (2) distinguished indigenous culture and history, (3) remarkable phytodiversity, (4) existence of different tribes and castes, (5) differences in methods of medicinal plant collection, their processing, preparations, usage and storage, (6) ethnobotanical knowledge variations, (7) less chance of the exchange of cultural knowledge between the study area to other areas may be due to restricted movement of people because of their residences in remote and hilly areas, (8) absence of a proper system of documentation, sharing and conservation of folk knowledge, (9) least interest of the younger generation in folk knowledge and practices, (10) differences in plant parts used, diseases treated and recipes, such as our study area's preparation methods, are different from other areas of Pakistan for the same plant part and treated disease, and (11) ethnomedicinal use of plant in our study area may not be documented or published from other study areas.

During surveys, it was observed that local plant resources are severely threatened by forest fires in summer, overgrazing (nomadic and normal), overexploitation and mining activities. People living in the far-flung mountains of the area have no/or less modern healthcare system, so most people rely on medicinal plant, and unsustainable collection may drive the flora to extinction [129–131]. During our study, it was also unveiled that over time, important folk indigenous knowledge about plants was limited to older people only, as the younger people have less interest in folk knowledge and traditional practices due to transforming lifestyle and culture; this can be inferred from the informant’s knowledge by age, which showed informants 6.2%, ≤ 30 years of age.

## Conclusion

In summary, the current study reported the important ethnomedicinal plant practiced in veterinary and human healthcare by the local people of District Haripur, Pakistan. Like the rural population of other countries, the local people also rely on medicinal plant to treat livestock and human diseases may due to traditional culture, easy availability and cheaper sources. Comparative analysis of the present study and their matching with other studies from Pakistan may reflect the novel use of these plants, which can provide a base line for pharmacognostic studies. Scientific and experimental validation of traditional knowledge is necessary to ensure safety and efficacy; therefore, the phytochemical, toxicological and clinical studies on the documented flora are recommended for a better understanding. In the study area, ethnomedicinal plant are also under severe threats, and combined efforts should be made to secure both the plant resources and folk knowledge. In this regard, awareness campaigns, conservation efforts and pharmacological and applied research studies are required.

## Supplementary Information

**Additional file 1: File S1**. Sample of questionnaire used during field survey for obtaining ethnobotanical information

**Additional file 2: Fig. S1**. Description of the study area, Haripur District, Khyber Pakhtunkhwa, Pakistan. **Fig. S2**. Images of some ethnoveterinary medicinal plant of District Haripur. **Table S1**. Relationship between Relative frequency of citation (RFC) and Use Value (UV)

## Data Availability

All the data are in manuscript and supporting documents.

## Abbreviations

EVM: ethnoveterinary medicine
RFC: relative frequency of citation
FC: frequency citation
∑Ui: sum of uses
UV: use values
S: shrubs
H: herbs
C: climbers
T: trees
A: annual
B: biennial
P: perennial
RT: root
LE: leaf
ST: stem
FR: fruit
SH: shoot
WP: whole plant
BA: bark
SD: seed
RH: rhizome
FL: flower
GM: gum
RE: resin
BB: bulb
TW: twigs
PP: pulp
SP: spine
