# Supplementary material for: Exploration of ethnomedicinal plants and their practices in human and livestock healthcare in Haripur District, Khyber Pakhtunkhwa, Pakistan
Source: J Ethnobiol Ethnomed. 2021 Sep 8;17:55. doi: 10.1186/s13002-021-00480-x (PMC8424965; doi:10.1186/s13002-021-00480-x)
Supplement: Supplementary file 1 — Additional file 1: File S1. Sample of questionnaire used during field survey for obtaining ethnobotanical information [file 13002_2021_480_MOESM1_ESM.docx]

**Exploration of ethnomedicinal plants and their practices in human and livestock healthcare in District Haripur, Pakistan**

**Proforma of Ethnobotanical Research- Questionnaire**

| ***S No*** | ***Item Name/Question*** | ***Obtained data*** | ***Other information*** |
| --- | --- | --- | --- |
|  | Informant Name |  |  |
|  | Age |  |  |
|  | Gender |  |  |
|  | Education level |  |  |
|  | Profession |  |  |
|  | Tribe |  |  |
|  | Locality/collection site |  |  |
|  | How long you are living in the area? |  |  |
|  | Are you familiar with plant? |  |  |
|  | What is its local name? |  |  |
|  | Most common medicinal uses of plant? |  |  |
|  | What are other uses of plant? |  |  |
|  | Which part of plant is used as ethnomedicine? |  |  |
|  | What is use-form of recipe? |  |  |
|  | What is mode of use of plant? |  |  |
|  | What is amount /dose used of the plant? |  |  |
|  | What is total length of dose use? |  |  |
|  | What is commercial perspective of the plant? |  |  |
|  | From whom you got this knowledge of ethnomedicine of the plant? |  |  |
|  | How do you collect the plant? |  |  |
|  | What are associated threats for medicinal plants? |  |  |
|  | Any other information of the plant use? |  |  |
