# Supplementary material for: Exploration of ethnomedicinal plants and their practices in human and livestock healthcare in Haripur District, Khyber Pakhtunkhwa, Pakistan
Source: J Ethnobiol Ethnomed. 2021 Sep 8;17:55. doi: 10.1186/s13002-021-00480-x (PMC8424965; doi:10.1186/s13002-021-00480-x)
Supplement: Supplementary file 2 — Additional file 2: Fig. S1. Description of the study area, Haripur District, Khyber Pakhtunkhwa, Pakistan. Fig. S2. Images of some ethnoveterinary medicinal plant of District Haripur. Table S1. Relationship between Relative frequency of citation (RFC) and Use Value (UV) [file 13002_2021_480_MOESM2_ESM.docx]

**
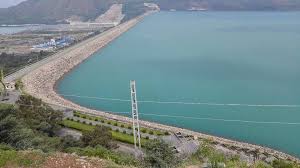

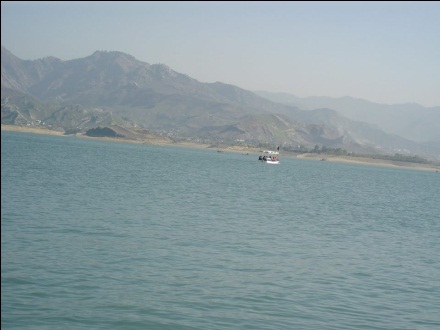

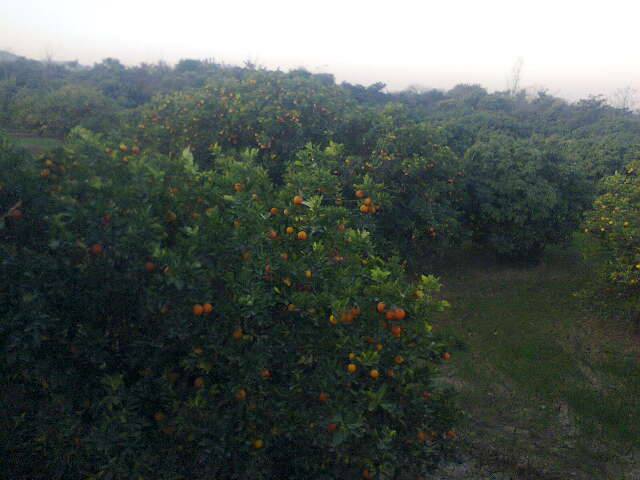
**

Tarbela dam Khanpur Dam Orange fields

**
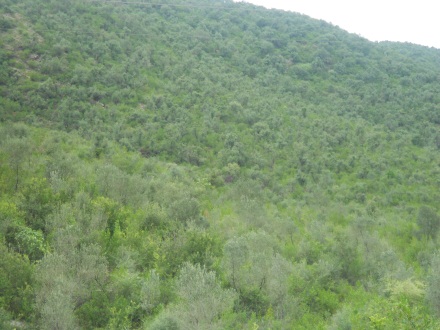

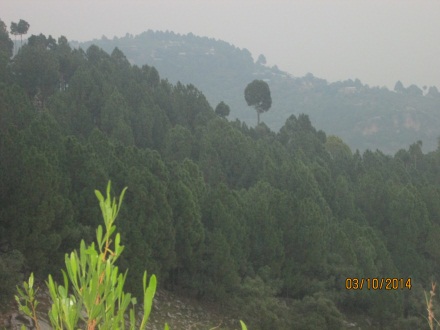

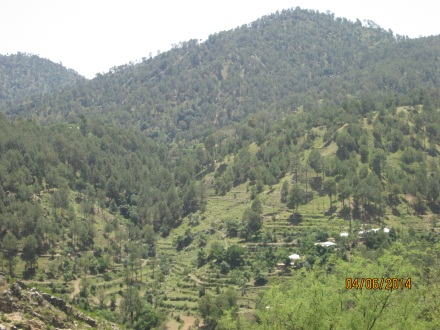
**

“*Kaho*” & “*Phulai*” forest Thick “*Chir*” forest A village in the study area

**
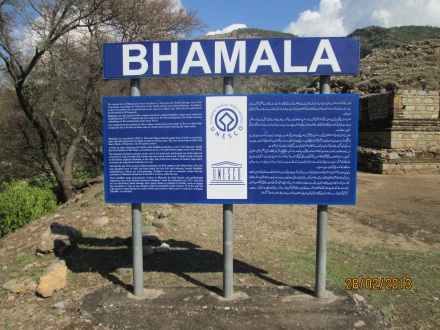

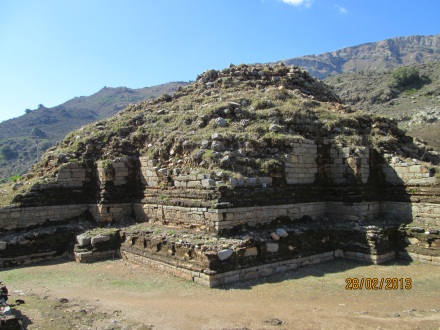

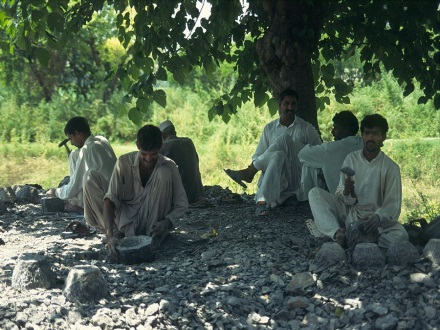
**

Bhamala Stupa (an ancient Gandhara Civilization remains) & traditional stone workers

**S1 Fig.** Description of the study area, Haripur District, Khyber Pakhtunkhwa, Pakistan


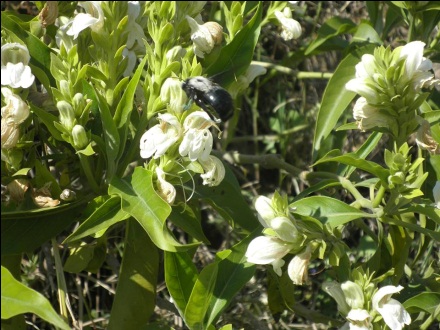

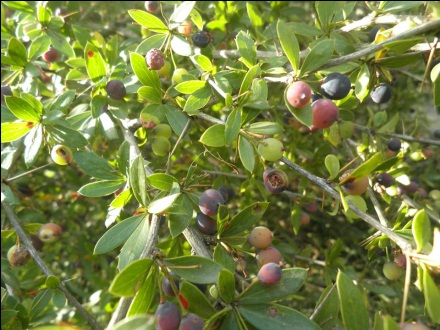

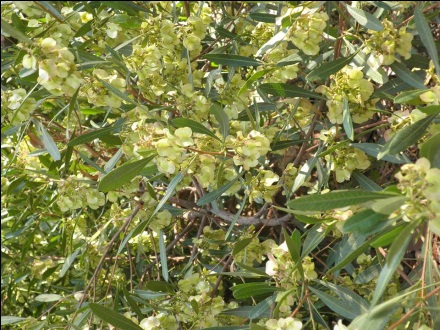


*Adhatoda vasica* Nees *Berberis lycium* Royle *Dodonaea viscosa* (L.) Jacq.


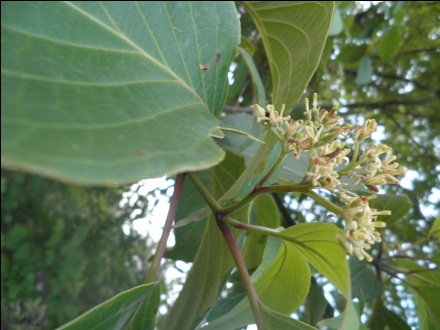

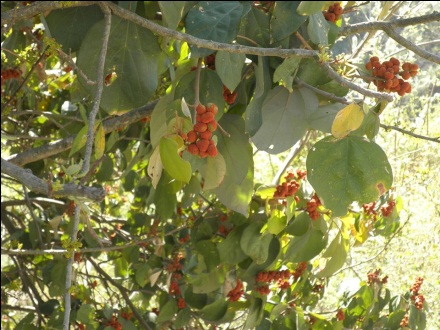

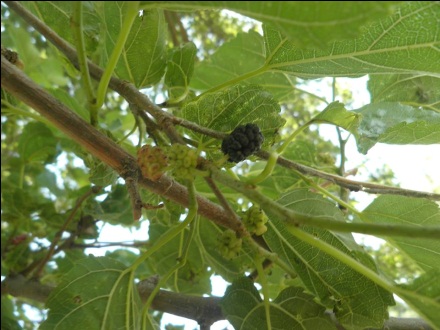


*Ficus benghalensis* L. *Mallotus philippinensis* Muell. *Morus nigra* L.


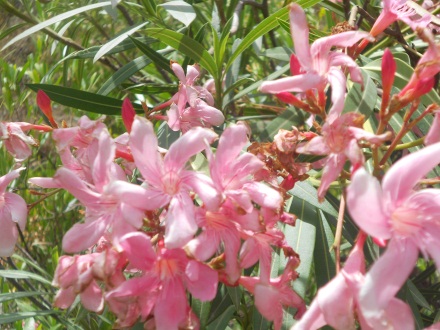

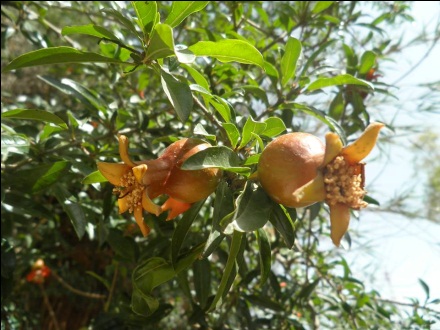

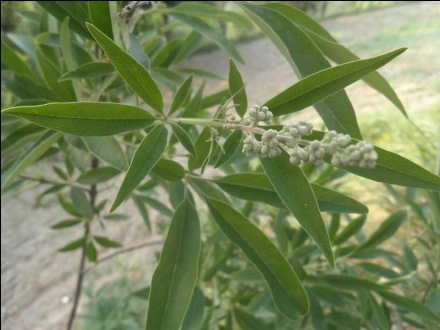


*Nerium Indicum* L. *Punica granatum* L. *Vitex negundo* L.

**S2 Fig.** Images of some ethnoveterinary medicinal plants of District Haripur

**S1 Table.** Summary Stats for Relative Frequency Citation (RFC) and Use Value (UV)

|  | **Correlations** | RFC | UV |
| --- | --- | --- | --- |
| RFC | Pearson Correlation | 1 | .371^**^ |
|  | Sig. (2-tailed) |  | .001 |
|  | N | 80 | 80 |
| UV | Pearson Correlation | .371^**^ | 1 |
|  | Sig. (2-tailed) | .001 |  |
|  | N | 80 | 80 |
| **. Correlation is significant at the 0.01 level (2-tailed). | | | |
